# Angiomyxoma in Accessory Hepatic Lobe

**Published:** 2016-01-01

**Authors:** Alejandro Rossi, Federica Pederiva, Rui Santos, Sarah Wood, Gill Humphrey

**Affiliations:** 1 Department of Paediatric Surgery, Royal Manchester Children’s Hospital, Oxford Road, Manchester M13 9WL, UK; 2 Department of Paediatric Radiology, Royal Manchester Children’s Hospital, Oxford Road, Manchester M13 9WL, UK

A 9-week-old female was referred with a 7-week history of increasing abdominal distension and non-bilious vomiting. Antenatal and postnatal history was unremarkable. Physical examination revealed a large palpable mass filling most of abdomen, with no jaundice. Results of laboratory and tumour markers (serum β-HCG and α-fetoprotein) were within normal range. Abdominal ultrasound and CT-scan confirmed a homogenous cystic mass that filled most of the abdomen displacing solid viscera and bowel superiorly and posteriorly (Fig. 1).

**Figure:**
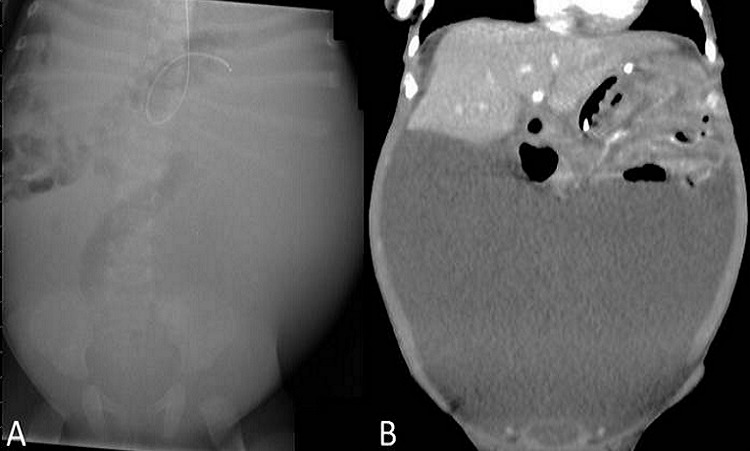
Figure 1: A: Abdominal x-ray showed a large opacity in the left side of the abdomen with associated displacement of bowel around it. B: In coronal CT scan a homogenous cystic mass was found filling most of the abdomen and displacing solid viscera and bowel.

At surgery, a large cyst attached to the inferior part of the accessory hepatic lobe was found. The cyst was removed together with the accessory lobe. The postoperative course was uneventful and the patient was discharged seven days after the procedure. Histopathological examination revealed a non-complicated hepatic angiomyxoma. No recurrence of tumour was seen on follow-up CT scan.

## DISCUSSION

Angiomyxoma is a rare (0.0017% in the general population), slowly growing, and benign proliferative mesenchymal tumor arising primarily in the soft tissue of the pelvis and perineum of adults. There is lack of agreement among pathologist regarding its pathogenesis; however, a fibroblastic/myofibroblastic origin seems most likely. Histologically, it consists of spindle-shaped or stellate cells widely separated by loose, myxoid stroma, and a prominent vascular component. Most patients are asymptomatic however others may complain of pain or abdominal distension and pressure symptoms. The diagnosis can be difficult. Ultrasound, CT-scan, and MRI can be useful tests to help in diagnosis. The main treatment is surgery with the aim to remove the whole tumour. Despite local recurrence reaching up to 47%, the prognosis is considered good.[1] Ectopic liver and hepatic accessory lobes are extremely rare congenital anomalies (0.09-0.7%), which can be associated with benign tumours.[2-4]

## Footnotes

**Source of Support:** Nil

**Conflict of Interest:** None declared

